# Structural Insight into the Mechanism of σ32-Mediated Transcription Initiation of Bacterial RNA Polymerase

**DOI:** 10.3390/biom13050738

**Published:** 2023-04-25

**Authors:** Qiang Lu, Taiyu Chen, Jiening Wang, Feng Wang, Wenlong Ye, Lixin Ma, Shan Wu

**Affiliations:** State Key Laboratory of Biocatalysis and Enzyme Engineering, Hubei Collaborative Innovation Center for Green Transformation of Bio-Resources, Hubei Key Laboratory of Industrial Biotechnology, School of Life Sciences, Hubei University, Wuhan 430062, China; luqiang@stu.hubu.edu.cn (Q.L.);

**Keywords:** Cryo-EM, transcription initiation, RNAP, σ^32^

## Abstract

Bacterial RNA polymerases (RNAP) form distinct holoenzymes with different σ factors to initiate diverse gene expression programs. In this study, we report a cryo-EM structure at 2.49 Å of RNA polymerase transcription complex containing a temperature-sensitive bacterial σ factor, σ^32^ (σ^32^-RPo). The structure of σ^32^-RPo reveals key interactions essential for the assembly of *E. coli* σ^32^-RNAP holoenzyme and for promoter recognition and unwinding by σ^32^. Specifically, a weak interaction between σ^32^ and −35/−10 spacer is mediated by T128 and K130 in σ^32^. A histidine in σ^32^, rather than a tryptophan in σ^70^, acts as a wedge to separate the base pair at the upstream junction of the transcription bubble, highlighting the differential promoter-melting capability of different residue combinations. Structure superimposition revealed relatively different orientations between βFTH and σ_4_ from other σ-engaged RNAPs and biochemical data suggest that a biased σ_4_–βFTH configuration may be adopted to modulate binding affinity to promoter so as to orchestrate the recognition and regulation of different promoters. Collectively, these unique structural features advance our understanding of the mechanism of transcription initiation mediated by different σ factors.

## 1. Introduction

Initiation of DNA transcription is one of the main regulatory steps in the control of gene expression. During bacterial transcription initiation, σ factors associate with the RNAP core enzyme, guide the transcription machinery to promoter regions of genes, and engage double-strand promoter DNA [1,2]. The resulting transition from closed RNAP–promoter complex (RPc) to open RNAP–promoter complex (RPo) with melted base pairs (bp) leads to de novo RNA synthesis. Different elements in the promoter DNA, including the −35, extended −10, −10, and discriminator elements, engage intensive interaction with σ factors. Primary σ factor (group 1) is responsible for transcription of housekeeping genes, while alternative σ factors (group 2, group 3, and group 4) control transcription of genes during adaptation to certain intracellular and environmental signals [3,4]. One group of σ factors belonging to the σ^54^ family is involved in nitrogen metabolism and certain stress responses [5,6].

The group 1 factors, represented by σ^70^, are the most studied and well-known factors. *E. coli* σ^70^ is composed of multiple domains, including σ_1.1_, σ_1.2_, σ_NCR_, σ_2_, σ_3.1_, σ_3.2_ and σ_4_. σ_1.1_ serves as a gatekeeper, admitting the promoter in and affecting the switching of RNAP properties [7,8,9]. Σ_1.2_ is responsible for discriminator motif recognition [10]. Σ_NCR_ participates in DNA interaction around the transcription start site [11]. Σ_3.1_ plays a role in “extended −10 motif” recognition and promoter binding [12,13]. Σ_3.2_ threads the RNAP and makes extensive interactions in the active center cleft, thereby affecting primer binding and promoter escape [14,15,16]. σ_2_ contacts the clamp helices of the β’ subunit and mediates sequence-specific interactions with the promoter −10 element, while σ_4_ contacts the β-flap tip helix (βFTH) and mediates sequence-specific interaction with the promoter −35 element [17]. In contrast, group 2 σ factors (such as *E. coli* σ^38^) lack the σ_NCR_ domain, group 3 σ factors (such as *E. coli* σ^28^ and σ^32^) lack σ_1.1_, σ_NCR_, and σ_1.2_ domains, and group 4 σ factors (such as *E. coli* σ^24^ and σ^19^) contain only two essential domains: σ_2_ and σ_4_.

The group 3 σ factor, σ^32^, plays a pivotal role in the heat shock response (HSR) required by *E. coli* to sustain protein homeostasis and cope with heat and other stresses [18]. σ^32^ synthesis is modulated by the secondary structure of the 5′ region of σ^32^ mRNA, which serves as a “thermometer”, responding to temperature changes [19,20]. When the temperature for growing *E. coli* cells suddenly increases, σ^32^ is rapidly synthesized (induction phase) and then directs RNA polymerase to promote transcription of a set of HSR genes to respond to environmental temperature changes [21]. The overproduced σ^32^ is gradually decreased by a set of conserved chaperones (such as DnaK/DnaJ) and proteases that accumulate during the induction phase [22,23].

In *E. coli*, the transcription initiation complex structures of σ^70^ [11,24], σ^38^ [25], σ^28^ [26], σ^24^ [15], and σ^54^ [27,28,29] have greatly advanced our understanding of how σ factors recognize corresponding promoter elements and initiate transcription. However, the structure of the RNAP holoenzyme or transcription initiation complex that interacts with group 3 σ^32^ or group 4 σ^19^ remains unknown. In this study, we assembled the functional *E. coli* transcribing complex with σ^32^ and determined the structure at 2.49 Å resolution. The structure shows that σ^32^ intensively interacts with promoter −10 and −35 element. Furthermore, several unique structural features were observed and the roles of key amino acids involved were validated through mutagenesis studies.

## 2. Materials and Methods

### 2.1. E. coli σ^32^ or σ^32^ Derivatives

*E. coli* strain BL21(DE3) (Novagen, Beijing, China) was transformed with plasmid pET28a-rpoH. Single colonies of the resulting transformants were used to inoculate 50 mL LB broth containing 50 μg/mL Kanamycin and cultures were incubated, with shaking, for 16 h at 37 °C. Aliquots (10 mL) were used to inoculate 1 L LB broth containing 50 μg/mL Kanamycin and cultures were incubated, with shaking, at 37 °C until OD_600_ = 0.6. The cultures were induced by adding IPTG to 1 mM and incubated for 3 h at 37 °C. Then, the cells were harvested by centrifugation (5000 rpm for 10 min at 4 °C), resuspended in 20 mL buffer A (20 mM Tris–HCl, pH 8.0; 0.1 M NaCl; 1.5% glycerol) and lysed using a JN-02C cell disrupter (JNBIO, Guangzhou, China). The lysate was centrifuged (12,000 rpm for 45 min at 4 °C) and the supernatant loaded onto a 2 mL column of Ni-NTA agarose (Qiagen, Wuhan, China) equilibrated with buffer A. The column was washed with 10 mL buffer A containing 0.16 M imidazole and eluted with 10 mL buffer A containing 0.5 M imidazole. The sample was further purified using a 5 mL column of HiTrap Heparin HP (GE Healthcare, Boston, MA, USA) equilibrated in buffer A and eluted using a 100 mL linear gradient of 0.2–1 M NaCl in buffer A. Fractions containing *E. coli* σ^32^ or σ^32^ derivatives were pooled and stored at −80 °C. 

### 2.2. E. coli RNAP Core Enzyme

*E. coli* RNAP core enzyme was prepared from *E. coli* strain BL21(DE3) (Invitrogen, Inc., Carlsbad, CA, USA) transformed with plasmid pET28a-rpoA-rpoB-rpoC-rpoZ similar to pIA900 [30]. Single colonies of the resulting transformants were used to inoculate 50 mL LB broth containing 50 μg/mL Kanamycin, and cultures were incubated, with shaking, for 16 h at 37 °C. Aliquots (10 mL) were used to inoculate 1 L LB broth containing 50 μg/mL Kanamycin, cultures were incubated at 37 °C, with shaking, until OD_600_ = 0.6 and then induced by adding IPTG to 1 mM. The cultures were incubated for 3 h at 37 °C. The cells were subsequently harvested by centrifugation (5000× *g* for 10 min at 4 °C), resuspended in 20 mL lysis buffer (50 mM Tris-HCl, pH 7.9; 0.2 M NaCl; 2 mM EDTA; 5% glycerol; and 5 mM DTT), and then lysed using a JN-02C cell disrupter. After poly-ethyleneimine precipitation and ammonium sulfate precipitation, the pellet was resuspended in buffer B (10 mM Tris-HCl, pH 7.9; 0.5 M NaCl; and 5% glycerol) and loaded onto a 5 mL column of Ni-NTA agarose equilibrated with buffer B. The column was washed with 25 mL buffer B containing 20 mM imidazole and eluted with 25 mL buffer B containing 0.15 M imidazole. The eluate was diluted in buffer C (10 mM Tris-HCl, pH 7.9; 0.2 M NaCl; and 5% glycerol) and loaded onto a Mono Q 5/10 GL column (GE Healthcare, Boston, MA, USA) equilibrated in buffer C and eluted using a 30 mL linear gradient of 0.3–0.5 M NaCl in buffer C. Fractions containing *E. coli* RNAP core enzyme were pooled and stored at −80 °C. 

### 2.3. Nucleic Acid Scaffolds and E. coli σ^32^-RPo Assembly

Nucleic acid scaffolds of the σ^32^ consensus promoter dnaKp1 for cryo-EM study of *E. coli* σ^32^-RPo and for fluorescence polarization study were prepared from synthetic oligos (F: 5′-CCCCCTTGAAGACGTGGTTTACGACCCCATTTAGTAGTCAACCGCAGTGAGTGA-3′; R: 5′-TCACTCACTGCGGTTGACTACTAAATGGGGTCGTAAACCACGTCTTCAAGGGGG-3′) [31] using an annealing procedure (95 °C for 5 min followed by 2 °C step cooling to 25 °C) in annealing buffer (5 mM Tris-HCl, pH 8.0; 200 mM NaCl; and 10 mM MgCl_2_).

*E. coli σ^32^-RPo,* the *E. coli* RNAP core enzyme, *E. coli* σ^32^, and nucleic acid scaffolds were mixed at 1:4:1.2 molar ratios and incubated at 4 °C overnight. The RPo complexes were purified using a Hiload 16/60 Superdex 200 column (GE Healthcare, Boston, MA, USA) and stored in 20 mM Tris–HCl pH 8.0, 0.1 M NaCl, 1% glycerol, and 1 mM DTT with a concentration of 5 mg/mL.

### 2.4. In Vitro Transcription Assay

In vitro Mango-based transcription assays [32] were carried out by incubating the *E. coli* RNAP core enzyme, *E. coli* σ^32^ or σ^32^ derivatives, and dnaKp1-mango scaffold. Transcription assays were performed using a 96-well microplate format. Reaction mixtures (40 µL) contained 0.1 μM *E. coli* RNAP holoenzyme, 0.1 μM *E. coli* σ^32^ or σ^32^ derivatives, 50 nM dnaKp1-mango scaffold, 1 μM TO1-Biotin, 0.1 mM NTP mix (ATP, UTP, GTP and CTP), 40 mM Tris–HCl, pH 8.0, 50 mM NaCl, and 10 mM MgCl_2_. First, the *E. coli* RNAP core enzyme, *E. coli* σ^32^ or σ^32^ derivatives, and dnaKp1-mango scaffold were incubated for 10 min at 37 °C. Following incubation with NTP mix and TO1-biotin for another 10 min at 37 °C, fluorescence emission intensities were measured using a multimode plate reader (EnVision, PerkinElmer Inc., Waltham, MA, USA; excitation wavelength = 510 nm; emission wavelength = 535 nm). Relative transcription activities of σ^32^ derivatives were calculated as described in a previous report [32]. 

### 2.5. Fluorescence Polarization Assay

**Figure 1:**
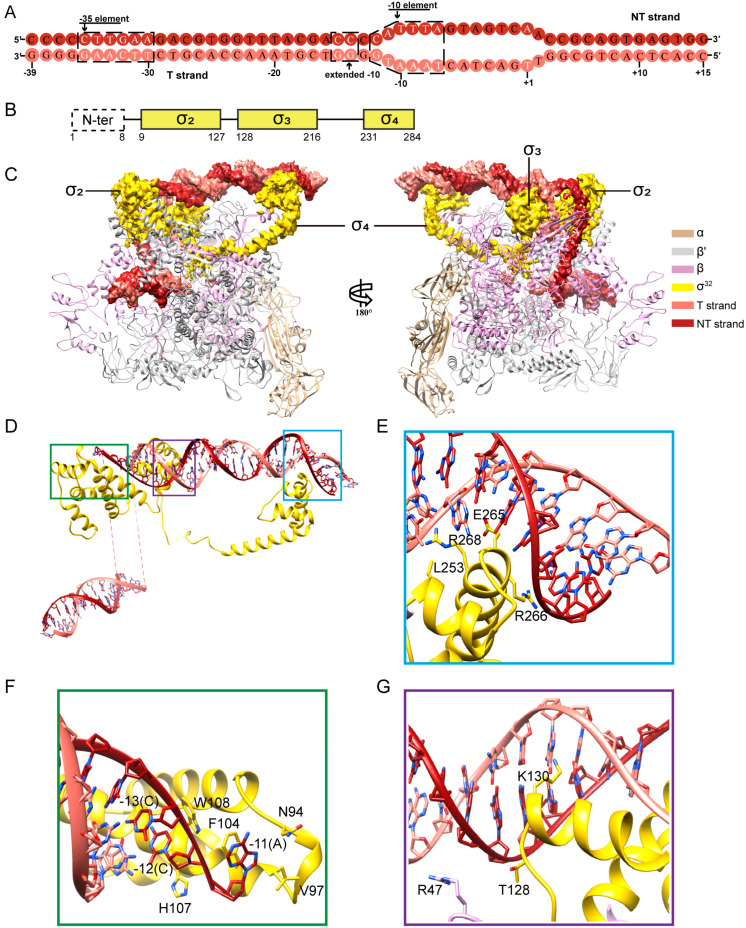
Cryo-EM structure of the *E. coli* σ^32^-RPo complex and interaction between σ^32^ and promoter DNA. (**A**) Nucleic acid scaffold. A schematic representation of the synthetic promoter DNA scaffold (54-bp) in the σ^32^-RPo. The −35, −10, and the extended −10 elements are annotated by a dashed box. Positions are numbered relative to the transcription start site. (**B**) Domain architecture of σ^32^, N-ter fragment of σ^32^ in the empty frame was not modeled in our structure. (**C**) Overviews of the structure and partial cryo-EM reconstruction maps of the *E. coli* σ^32^-RPo. The subunits of β’, β, α, T strand, NT strand, and σ^32^ are colored light brown, purple, gray, yellow, hot pink, and maroon, respectively. The individual domain of σ^32^ is labeled. (**D**) Overall interaction between σ^32^ and promoter DNA. The dashed boxes indicate the interaction regions. (**E**) Detailed interaction between σ_4_ and −35 element of promoter DNA. (**F**) Detailed interaction between σ_2_ and −10 element of promoter DNA. (**G**) Detailed interaction between σ_3_ and −35/−10 spacer region of promoter DNA.

**Figure 2:**
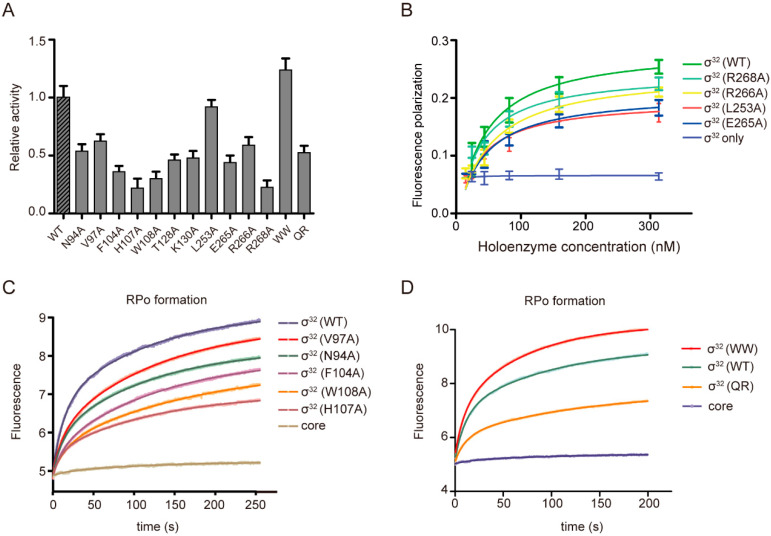
In vitro transcription activity, DNA binding affinity, and promoter unwinding activity of σ^32^ and σ^32^ derivatives. (**A**) In vitro transcription activity of σ^32^ and σ^32^ derivatives. The experiments were conducted in triplicate (mean± SEM; 3 determinations). Error bars represent mean± SEM of n = 3 experiments. (**B**) Binding affinities of wild-type (WT) σ^32^ or σ^32^ derivatives holoenzyme and promoter −35 element measured using fluorescence polarization (FP) assay. (**C**) Promoter unwinding activity of wild-type (WT) σ^32^ or σ^32^ derivatives holoenzyme (σ^32^ derivatives in the “A-11” pocket) measured using stopped-flow assay. (**D**) Promoter unwinding activity of σ^WW^, σ^HW^ (WT), and σ^QR^ measured using stopped-flow assay.

**Figure 3:**
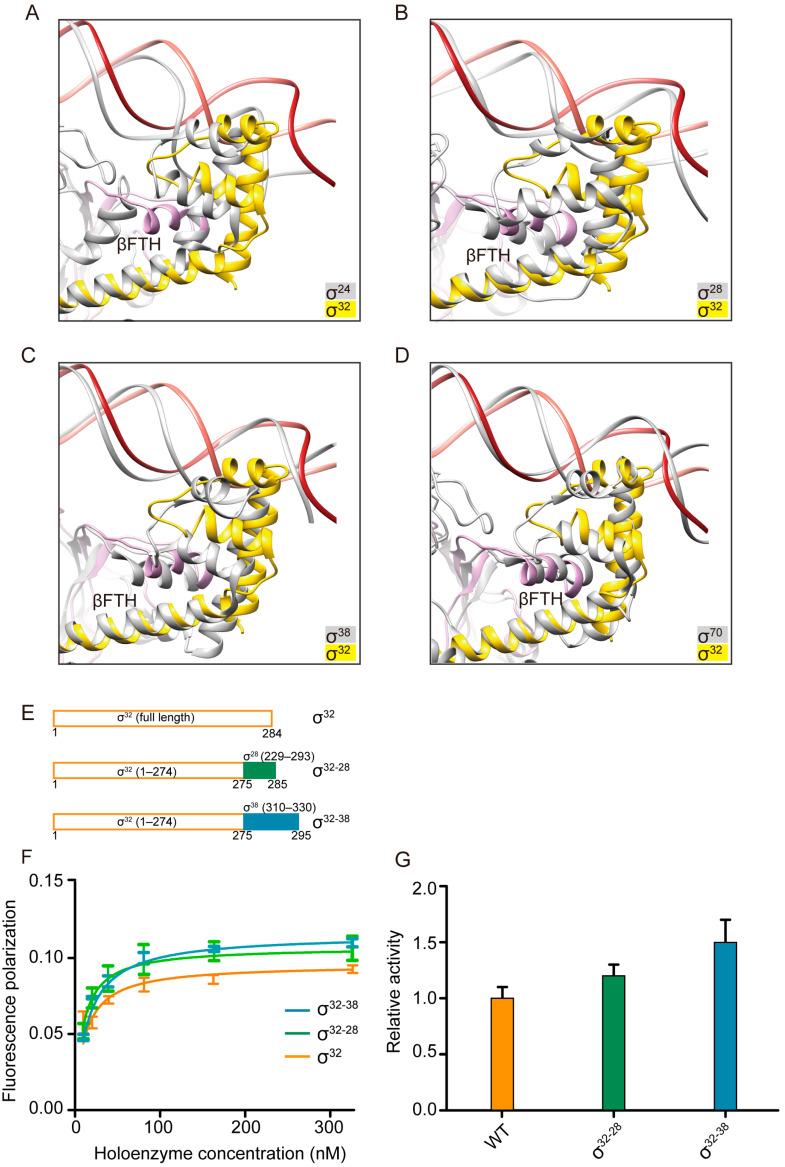
Superimposition and characteristics of βFTH–σ_4_ interactions. (**A**–**D**) Superimposition of βFTH–σ_4_ interactions between σ^32^ and σ^24^ (PDB: 6JBQ), σ^28^ (PDB: 6PMI), σ^38^ (PDB: 6OMF), and σ^70^ (PDB: 6CAO), respectively. (**E**) Schematic diagram of the WT, chimera σ^32–28^, and chimera σ^32–38^. (**F**) Binding affinities of wild-type σ^32^ or σ^32^ derivative holoenzyme and promoter measured using the FP assay. (**G**) In vitro transcription activities of WT, chimera σ^32–28^, and chimera σ^32–38^ (mean ± SEM; 3 determinations). Error bars represent mean± SEM of n = 3 experiments.

We measured the affinity of two FAM-labeled holoenzyme DNA molecules. The shorter DNA (containing the −35 element) was used to validate the role of DNA-contacted amino acids in σ_4_ (Figure 1E and Figure 2B), while the longer DNA (containing the sequence from the −35 element to the −10 bubble) was used to validate the effect of σ_4_–βFTH configuration changes on affinity (Figure 3F). The sequence of the shorter 6-FAM-labeled DNA template strand was 5′-CCTTGAAGACGTGG-3′ and the nontemplate strand was 5′-CCACGTCTTCAAGG-3′; the sequence of the longer 6-FAM-labeled DNA template strand was 5′-CCTTGAAGACGTGGTTTACGACCCCATTTAGTAG-3′ and the nontemplate strand was 5′-GGGGTCGTAAACCACGTCTTCAAGG-3′. The labeled template strand and the nontemplate strand were annealed in a 1:1 ratio in 10 mM Tris-HCl, pH 7.9 and 0.2 M NaCl and stored at −80 °C. The annealed DNA (5 nM) was incubated with *E. coli* σ^32^ or σ^32^ derivatives holoenzyme (10, 20, 40, 80, 160, and 320 nM) in 100 μL FP-A buffer (10 mM Tris-HCl, pH 7.7; 100 mM NaCl; 1 mM DTT; 1% glycerol; and 0.025% Tween-20) in a 96-well plate (Corning, NYC, USA) for 5 min at room temperature. The fluorescence polarization (FP) signals were measured using a plate reader (SPARK, TECAN, Switzerland) at excitation wavelength = 494 nm and emission wavelength = 518 nm. The calculated Kd is an apparent Kd obtained from the best-fit curve by fitting our plots to a nonlinear regression equation [33,34].

Fluorescence polarization was calculated using the formula:

P = (I_VV_ − I_VH_)/(I_VV_ + I_VH_)

where I_VV_ and I_VH_ are fluorescence intensities with the excitation polarizer at the vertical position and the emission polarizer at the vertical position and the horizontal position, respectively. The equilibrium dissociation constant, Kd, was calculated using the equation:

P = P_f_ + {(P_b_ − P_f_) × [T]/(Kd + [T])}

where P is the fluorescence polarization at a given concentration of holoenzyme, P_f_ is the fluorescence polarization of free 6-FAM-labeled promoter DNA, P_b_ is the fluorescence polarization of bound 6-FAM-labeled promoter DNA, and [T] is the concentration of holoenzyme harboring WT or mutant σ^32^.

### 2.6. Stopped-Flow Assay

The promoter for the stop flow assay was prepared as previously reported [15]. The Cy3-amido-dT-modified nontemplate-strand primer (*dnaKp1*-Cy3-nontemplate: 5′-GCATCTCCCCCTTGAAGACGTGGTTTACGACCCCATTTAGTAGTC/iCy3dT/ACCGCAGT-3′; 10 µM, Sangon Biotech) and the template-strand primer (*dnaKp1*-template: 5′-GGCACGTACGAATATACCACATACCAATCCTTCCTTCGTACGTGCACTCACTCACTGCGGTTGACTACTAAAT-3′; 10 µM, Sangon Biotech) were processed as previously reported [15]. To monitor the efficiency of RPo formation of *E. coli* RNAP holoenzymes comprising wild-type or derivatives of *E. coli* σ^32^, 60 µL σ^32^-RNAP holoenzyme (200 nM) and 60 µL Cy3- PdnaKp1 (4 nM) in 10 mM Tris–HCl (pH 7.7), 20 mM NaCl, 10 mM MgCl_2_, and 1 mM DTT were rapidly mixed and the change in Cy3 fluorescence was monitored in real time using a stopped-flow instrument (SX20, Applied Photophysics Ltd., UK) equipped with an excitation filter (515/9.3 nm) and a long-pass emission filter (570 nm). The data were plotted in SigmaPlot (Systat software, San Jose, CA, USA) and fitted to eqn: F = F_0_ + *a*_1_ × (1 − exp × (−*k_obs,_*_1_ × t)) + *a*_2_× (1 − exp × (−*k_obs,_*_2_ × t)). The amplitudes (*a*_1_ and *a*_2_) and observed rates (*k_obs,_*_1_ and *k_obs,_*_2_) were estimated as previously reported [15,35].

### 2.7. Cryo-EM Grid Preparation

Grids (Quantifoil 1.2/1.3 Au 300 mesh) were glow-discharged for 25 s at 15 mA prior to application of 4 μL complex at a concentration of 1.2 mg/mL, then plunge-frozen in liquid ethane using a Vitrobot (FEI, Valley, SD, USA.) with 100% chamber humidity at 4 °C.

### 2.8. Cryo-EM Data Acquisition and Processing

Grids were transferred to a 300 kV Titan Krios electron microscope equipped with a Gatan K3 summit direct electron detector and a GIF energy filter with a slit width of 20 eV. All the images were auto-collected via EPU [36] with a magnification of 105 k and exposure time of 2.5 s using a 0.851 Å pixel size and a defocus ranging from −1.0 to−1.5 μm. The total dose was about 54 e/Å^2^. A total of 8555 movies were imported and processed using Relion. All 40 frames were aligned using MotionCor2 [37] before contrast transfer function (CTF) estimations. The contrast transfer function was estimated for each summed image using CTFFIND4 [38]. From the summed images, ~4,000,000 particles were auto-picked and subjected to 2D classification in RELION [39]. 2D averages of the best classes were used as templates for auto-picking in RELION. Auto-picked particles were manually inspected and then subjected to 2D classification in RELION. Poorly populated classes were removed, resulting in a dataset of 641,734 particles. These particles were re-extracted without binning and processed further using 3D refinement and CTF refinement. The final density map was post-processed in RELION with a resolution of 2.49 Å. Focus refinement with mask and signal subtract improved local resolution of the DNA-σ^32^ interaction area to 3.40 Å.

### 2.9. Cryo-EM Model Building and Refinement

The coordinates of core RNAP and promoter DNA from the structure of *E. coli* core RNAP (PDB: 7MKP) [40] were fitted into the cryo-EM density map using chimera [41] and manually modified in COOT [42]. The coordinates of σ^32^ were manually built in COOT [42]. The coordinates were real-space refined with secondary structure restraints in Phenix [43]. Due to the weak map signal, the region between the −10 bubble and downstream double-stranded DNA was not modeled. The portions of chains C (residues 233–332 and 974–1025) and F (residues 960–1126), which are in low-resolution maps and not the main focus of our work, were also not modeled.

## 3. Results

### 3.1. The Cryo-EM Structure of E. coli σ^32^-RPo

To determine the structure of *E. coli* σ^32^-RPo, we reconstituted the complex with the *E. coli* RNAP core enzyme, σ^32^, and a nucleic acid scaffold (Figure 1). The nucleic acid scaffold (−39 to +15; +1 as transcription start site) is composed of a 28-bp upstream double-stranded DNA (dsDNA) with consensus sequences of the −35 element, a 13-bp transcription bubble (complementary sequences on both strands), and a 13-bp downstream dsDNA (Figure 1A).

We obtained a cryo-EM map at 3.0 Å for the *E. coli* σ^32^-RPo complex with local resolution at the active-center cleft of RNAP around 2.49 Å (Figure 1C and Figure S1 and Table 1). The map shows clear densities for residues of σ_2_ (residues 47–127) and σ_4_ (residues 231–279) (Figure 1B,C). The map also shows clear densities for the upstream part of the transcription bubble and the downstream dsDNA (Figure 1C,D). Similar to studies on other σ factors [11,44], the overall structure of RNAP shows minimal structural change upon σ^32^ engagement. Structural superimposition between RNAP core (PDB 7MKP) and σ^32^-RNAP results in an RMSD of 1.633 Å across 3268 residues [45]. Similar to other σ factors, the RNAP clamp in the structure of σ^32^-RPo remains in a closed conformation (Figure 1C). The omega subunit is absent from the solved structure, probably due to low binding affinity.

In the structure of σ^32^-RPo, the σ_2_ and σ_4_ domains bind to the surface of RNAP (Figure 1C). The σ_2_ domain attaches to the clamp helices of the β′ subunit of RNAP via a polar surface. The interface residues include D77, E81, I84, and M87 of σ^32^, and R275, R278, R281, L282, L285, I291, E295, and M298 of clamp helix of the β′ subunit (Figure S3A). The σ_4_ domain of σ^32^ uses a distinct hydrophobic surface to bind the βFTH of RNAP. The interface residues include A221, L225, W244, L245, L278 and E283 of the σ_4_ domain and E898, K900, L901, I905 and F906 of the βFTH (Figure S3B). Furthermore, σ^32^ strongly interacts with different DNA elements in the promoter DNA (Figure 1D–G). The N-terminus of σ^32^ was not modeled in the structure, possibly due to the flexibility caused by the presence of intrinsically disordered regions.

### 3.2. The Interactions between σ_4_ Domain and the −35 Element

σ^32^-regulated promoters have a distinct consensus sequence at their −35 elements (5′-CTTGAA-3′; from −35 to −30). In the structure, σ_4_ adopts a helix-turn-helix fold (Figure 1D), inserts into the major groove of dsDNA around the −35 element, and potentially makes base-specific polar interactions with nucleotides (Figure 1E). The interactions are important, as substituting E265, R266, or R268 with alanine resulted in substantial loss of transcription activity (Figure 2A). The effect of L253A is modest compared with that of other residues. Furthermore, the fluorescence polarization assay shows that the substitution of these residues with alanine profoundly decreases binding affinity between σ^32^ and −35 element. The calculated K_D_ values of L253A, E265A, R266A, and R268A mutants are 38.75 nM, 45.88 nM, 54.87 nM, and 46.72 nM, respectively, while the K_D_ value of the wild-type σ factor is 28.41 nM, justifying the functional importance of these interactions (Figure 2B). It is worth noting that while σ^32^-RNAP holoenzyme readily interacts with promoter −35 element DNA, the sole σ^32^ fails to bind DNA (Figure 2B), which is consistent with previous reports that sole σ subunits adopt a closed conformation and are incapable of binding DNA [46,47].

### 3.3. The Interactions between σ^32^ and −35/−10 Spacer

The interactions between σ factors and −35/−10 spacer were established to stabilize the conformation of the upstream DNA duplex, promoting the engagement of the upstream duplex with σ_4_ and σ_2_ for subsequent promoter unwinding. The σ^32^-RPo complex contacts the phosphate backbones of the spacer region between the −35 and the −10 elements at three positions (Figure 1D). Beside the reported interaction between R47 of the RNAP-β′ zipper domain and phosphate backbones of nucleotides between G_−17_ and C_−18_ on the nontemplate DNA strand, σ^32^ was also found to interact with −35/−10 spacer. Specifically, T128 contacts the same phosphate backbones of nucleotides between G_−17_ and C_−18_. K130 of σ^32^ makes a H-bond with N7 atoms of G_−15_ on the template DNA strand, which together with G_−14_ were identified to be the “extend −10” element [48] (Figure 1G). Substituting T128 and K130 with alanine causes a substantial loss of transcription activity (Figure 2A), highlighting the importance of these interactions [48].

### 3.4. The Promoter-DNA-Unwinding Function of σ^32^

σ^70^, σ^38^, and σ^28^ of *E. coli* unwind promoter DNA at the −11/−12 junction [26,44,49], while σ^24^ unwinds promoter DNA at a position 1 bp upstream of the transcription bubble [15]. Our structure shows that the C:G bases at position −12 are paired, while the A:T bases at position −11 are unwound (Figure 1F). This observation strongly suggests that σ_2_ unwinds promoter DNA at the −11/−12 junction, which is consistent with that of σ^70^ [49,50]. Specifically, H107 blocks the path upstream dsDNA and serves as a wedge to disrupt base stacking between positions A_−11_ and C_−12_ (Figure 1F). The unwound nucleotide A_−11_ on the nontemplate DNA strand is stabilized by a pocket created by N94, V97, F104, and W108 on the “specificity loop” of σ_2_ domain [51] (Figure 1F). A similar pocket is reported where different bases are recognized [11,15]. The functional importance of these residues in transcription was validated by our experiment, which showed that substitution with alanine resulted in significant loss of transcription activity (Figure 2A).

To explore the contributions of these residues in promoter unwinding, we modified a stopped-flow assay to monitor RPo formation by *E. coli* σ^32^-RNAP, in which the fluorescence of a Cy3 fluorophore at +1 position on the nontemplate DNA strand increases upon RPo formation. As shown in Figure 2C and Table S1, the kinetics of RPo equilibration of σ^32^ (H107A)-RNAP holoenzyme is significantly slower than that of wild-type σ^32^-RNAP. Mutations in the protein pockets of A_−11_ (N94A, V97A, W108A and F104A) also exhibited slowed RPo equilibration. It is worth noting that the curves could well fit to a double exponential where the amplitudes and observed rates for the fast (*a*_1_, *k_obs,_*_1_) and slow (*a*_2_, *k_obs,_*_2_) phases were fixed [35] (Table S1), suggesting the existence of a significant intermediate (RPi) on the path to RPo formation. The amplitudes (*a*_1_, *a*_2_) describe that phase’s contribution to the total fluorescent enhancement signal. As show in Table S1, the amplitude of the curve is dominated by the amplitude of the slow rate, which implies that the slow phase contributes more to final RPo formation. As fluorescence enhancement serves as a reporter of the open complex, we conclude that substituting these residues with alanine significantly destabilizes the open *E. coli* RNAP complex. Specifically, our results show that substituting W108, H107, and F104 with alanine greatly destabilizes the RPo, while substituting V97 and N94 with alanine only slightly destabilizes the RPo (Table S1).

Sequence alignment and structural analysis of other *E. coli* σ factors indicate that diverse residues lie in the same position in different σ factors and play the same role as H107–W108 of σ^32^ (Figures S2 and S4I–L). To compare the efficiency of these residues at promoter unwinding, we substituted the HW residues with WW (σ^70^ and σ^38^) and QR (σ^28^), and tested the corresponding transcription activity using a Mango-dependent in vitro transcription assay. As shown in Figure 2A, the rankings in transcription activity differ as follows: σ^WW^ > σ^HW^ (WT) > σ^QR^. Similarly, the RPo formation activity was ranked as σ^WW^ > σ^HW^ (WT) > σ^QR^ (Figure 2D, Table S2), indicating that distinct wedge residues were employed by different σ factors to orchestrate the recognition and unwinding of different promoters. We speculate that the HW motif in σ^32^ evolved from the WW motif in σ^70^ with the purpose of facilitating selective expression of different genes to adapt to changes in internal and external environments. One possibility is that the WH motif, beside its role in helping promoter DNA melting, and together with adjacent residues, may provide a unique structural feature that allows σ^32^ to interact with specific promoters and regulate the expression of heat shock genes.

Notably, the curves and parameters (*k_obs,_*_1_~0.1^s−1^, *k_obs,_*_2_~0.01^s−1^) in Figure 2C,D are similar and resemble those in *E. coli* σ^24^-RNAP [15] rather than those in *M. tuberculosis* CarD-RNAP [52], which means that the observed fast rates (*k_obs,_*_1_~1^s−1^) report on protein–DNA interaction is not related to opening (i.e., binding) and the observed slow rate (*k_obs,_*_2_~0.1^s−1^) is related to RPo equilibration. These results indicated the importance of RPo formation for transcription, and also highlighted the multiplicity of RPo equilibration in different bacteria.

### 3.5. βFTH Adopts a Distinct Conformation to Orchestrate σ^32^-Promoter Recognition

To date, structures of four σ factors that belong to the σ^70^ family have been resolved, including σ^70^ [11,50], σ^38^ [25,44], σ^28^ [26], and σ^24^ [15]. We compared these published structures to dissect more details about the mechanism of promoter recognition. Structure superimposition revealed that the relative orientation between βFTH and σ_4_ in σ^32^-engaged RNAP differ between these σ-engaged RNAPs (Figure 3A–D). To further explore the role of βFTH in σ-mediated DNA engagement, we designed two chimeric σ factors to validate DNA binding affinity to the corresponding holoenzymes. Considering that (1) σ^38^ recognizes promoters overlap with σ^70^ but σ^70^ is larger than the other σ factors; (2) σ^28^ and σ^38^ belong to the same group 3 category; and (3) the tail of σ^24^ did not observably interact with βFTH, we chose σ^28^ and σ^38^ as our research targets. The chimera of σ^32^ and σ^28^, designated as σ^32–28^, was designed by replacing the βFTH-interacting σ_4_ segment of σ^32^ with the βFTH-interacting σ_4_ segment of σ^28^. The chimera of σ^32^ and σ^38^, designated as σ^32–38^, was designed by replacing the βFTH-interacting σ_4_ region of σ^32^ with the βFTH-interacting σ_4_ region of σ^38^ (Figure 3E). The calculated K_D_ values of WT, chimera σ^32–28^, and chimera σ^32–38^ are 14.95 nM, 9.22 nM, 8.34 nM, respectively. Owing to the larger binding area, the binding affinity in this experiment is higher than that in Figure 2B, and σ^32–28^ and σ^32–38^ both enhanced the affinity of promoter DNA binding to the holoenzyme as shown by the result of the fluorescence polarization assay (Figure 3F). Similarly, both chimeras enhanced transcription activities (Figure 3G). These results indicate the importance of σ_4_–βFTH interaction in σ^32^-promoter recognition, and also highlighted the important role of βFTH in orchestrating the recognition of other σ promoters. Collectively, our structural and biochemical results suggest that biased σ_4_–βFTH configurations in different σs may be adopted to modulate binding affinity to promoters so as to orchestrate the recognition and regulation of different promoters.

## 4. Discussion

Bacterial cells sense and respond to different environmental conditions by regulating different expression regulons via activation of distinct σ-RNAP holoenzymes [53]. Certain RNAPs harboring group 1 and group 2 σ factors can transcribe the same promoter sets [54]. *E. coli* σ^70^ shares a high degree of protein sequence similarity with σ^38^ and prefers binding to similar DNA sequences at the −10 and −35 promoter elements [55]. In contrast, the protein sequences and target DNA sequence of other σ factors are less similar.

Our structure revealed that the recognition between σ^32^-RPo and −35 element closely resembles that of σ^24^-RPo [15] rather than that of σ^28^-RPo [26], despite the fact that both σ^32^ and σ^28^ belong to group 3 (Figure 1E and Figure S4). By comparison, σ^70^-RPo and σ^38^-RPo strongly interact with −35 element and the spacer region, with both having six to eight residues participating in the interactions (Figure S4C,D,G,H), while σ^24^-RPo interacts modestly with the −35 element and the spacer region, with four residues participating in the interactions (Figure S4A,E). It is worth noting that the interaction between σ^28^ and −35 element is the weakest (Figure S4B), consistent with the finding that β’ZBD is relocated to help strength the interaction with the −35 element [26]. Furthermore, our study revealed a relatively weak interaction between σ^32^ and −35/−10 spacer region mediated by T128 and K130 of σ^32^ (Figure 1G), which is in congruence with a previous study [48]. Collectively, these structural features are consistent with the fact that group 2 σs are more similar to housekeeping group 1 σs, and group 1 and group 2 σs account for the transcription of a majority of genes in bacteria [2].

Our structure may also help solve or explain some previous questions. Based on our structure, we speculate that phosphorylation of Y260 may inhibit σ^32^ activity [56] by attenuating the interaction between adjacent residues and the −35 element or by triggering an unpopular σ_4_–βFTH configurations. Our structure also connects a series of disordered regions in σ^32^ previously reported in research on HSR: residues 47–55, termed feedback control region [57,58,59], are located in σ_2.1_ and are partially protected by residues 57–66 of the clamp helix of β‘, which is the binding site for DnaKJ chaperones [60,61]. Sandwiched between σ_1.2_ and σ_3.0_, residues 122–144 (Region C) [62] and residues 263–284 (The C-terminal region) [63] are mainly responsible for binding with RNAP. In addition, as σ^32^ I54 (which was shown to play a crucial role in membrane-targeted instability of σ^32^ during HSR) [57] was fixed and protected by the helix of σ_2.2_ and the clamp helix of the β’ subunit, we speculate that cytoplasmic σ^32^ faces two mutually exclusive fates: either bind to RNAP to exert their active function or be inactivated through membrane localization.

From first engaging with promoter DNA to finally producing RNA, RNAP holoenzymes harboring promoters undergo several sequential conformational states, including promoter recognition (RPc), promoter melting (RPi), and bubble formation (RPo) [64]. Promoter melting is nucleated by the wedge residue, as shown in our study and in previous reports [15,65]. The hallmark of RPo maturation is the formation of an approximately 12-nt “bubble” around the promoter −10 element, which allows initial RNA synthesis and NTPs incorporation. W-dyad was found to wedge the transcription bubble in σ^70^. While the presence of W-dyad is common in group 1 σ factors [66,67], residues harboring bulky hydrophobic sidechains are favored at corresponding sites in other σ factors [67]. Given the robust DNA-melting capacity of group 1 σ factors, it was believed that the W-dyad would be optimal for supporting the upstream transcription bubble. Our structure shows that H107 in σ^32^ replaces W433 in the W-dyad in σ^70^ to wedge the upstream transcription bubble, while W108 in σ^32^ resembles W434 in the W-dyad of σ^70^. Our data also show that different σ factors employ distinct wedge residues in initiating transcription with the following activity ranking: WW > HW > QR. This result is consistent with the result of the stopped-flow assay. Our result is in congruence with previous hypothesis that the W-dyad is the optimal choice for wedging the upstream ds/ss junction of the transcription bubble [65], giving its powerful DNA-melting capacity. Other residues such as H, N, and Q (Figure 1F and Figure S4I–J) with inferior promoter melting abilities may allow alternative σ factors to form RPo at a slower rate (Figure 2C,D), fine-tuning their specificity [15]. Variations in the residues at this very position may lead to differences in DNA-melting capabilities of different σ factors, leading to differential transcription activity and gene regulation [68].

During transcription initiation, conserved σ_4_ contacts the βFTH and mediates recognition of the promoter −35 element [69]. Previous reports established that variations in spacer lengths greatly affects transcription activity and leads to the concurrent rotation of the σ_4_–βFTH module, which plays a significant role in promoter recognition [70,71,72]. However, whether the σ_4_–βFTH modules of different σs can allosterically affect promoter recognition has not been explored. Here, two chimeric σ factors (σ^32–28^ and σ^32–38^) with partial σ_4_ domain replaced were designed to test the impact on DNA binding affinity and transcription activity, and it was surprising to find variation in DNA binding affinity and enhanced transcription activity with the chimeric proteins (Figure 3F,G). These results indicated the importance of σ_4_–βFTH interaction in σ^32^-promoter recognition and also highlighted the important role of βFTH in orchestrating the recognition of other σ promoters. Although our study shows that the σ_4_–βFTH interaction affects transcription activity by influencing the binding affinity of σ_4_ to the promoter, it seems that other steps such as promoter escape and transcription pausing may also play a role [16,49]. Nevertheless, our study indicates that the σ_4_–βFTH interaction is tuned to balance the distinct transcription efficiencies of different σ factors.

In summary, our structure and supporting biochemical data identified a different promoter recognition and promoter melting mode for transcription initiation, including (1) a relatively weak interaction between σ^32^ and −35/−10 spacer that may account for less efficient promoter recognition [2]; (2) a histidine with inferior promoter melting ability that may allow σ^32^ to form RPo at a slower rate, fine-tuning its specificity; (3) a biased σ_4_–βFTH configuration to modulate the affinity of binding to the promoter so as to orchestrate the recognition and regulation of different promoters. Our work will deepen our understanding of transcription initiation mechanisms of different σ^32^ factors as well as the biological function of σ^32^.

## Figures and Tables

**Table 1:** Statistics of cryo-EM data and structure refinement.

Data Collection and Processing	RPo
Magnification	105,000
Voltage (kV)	300
Electron exposure (e^−^/Å^2^)	54
Defocus range (μm)	−1.0~−1.5
Pixel size (Å)	0.851
Symmetry Imposed	*C*1
Number of micrographs	8557
Initial particle projections (no.)	3,854,813
Final particle projections (no.)	641,734
Map resolution (Å)	2.49
FSC threshold	0.143
Map resolution range	2.3–7.0
**Refinement**	
Initial model used	PDB 7MKP
Model resolution (Å)	2.64
FSC threshold	0.143
Map sharpening B factor (Å^2^)	−10
**Model composition**	
Nonhydrogen atoms	28,255
Protein residues	3386
Nucleotides	83
**B factors (Å^2^)**	
Protein	8.12/205.39/64.84
Nucleic acids	94.01/275.47/208.74
**Validation**	
MolProbity score	1.54
Clashscore	6.05
Poor rotamers (%)	0.03
Ramachandran plot	0.00
Favored (%)	96.67
Allowed (%)	3.33
**Model to Map**	
Map CC	0.8358

## Data Availability

Structure coordinates and cryo-EM density map of σ^32^-RPo have been deposited in the Protein Data Bank and Electron Microscopy Data Bank under accession numbers 8HKC and EMD-34849, respectively.

## Supplementary Materials

The following supporting information can be downloaded at: https://www.mdpi.com/article/10.3390/biom13050738/s1, Figure S1: Purification of *E. coli* σ^32^-RPo complex and Cryo-EM of the *E. coli* σ^32^-RPo complex; Figure S2: Sequence alignment of σ factors; Figure S3: Structural and interacted details of pointed motifs; Figure S4: The detailed interactions between σs (σ^24^, σ^28^, σ^38^ and σ^70^) and corresponding promoters. Table S1: Kinetic parameters from Figure 2C of RPo formation by *E. coli* RNAP holoenzyme comprising wild-type or derivatives of *E. coli* σ^32^; Table S2: Kinetic parameters from Figure 2D of RPo formation by *E. coli* RNAP holoenzyme comprising wild-type or derivatives of *E. coli* σ^32^.
