# Supplementary material for: Structural Insight into the Mechanism of σ32-Mediated Transcription Initiation of Bacterial RNA Polymerase
Source: Biomolecules. 2023 Apr 25;13(5):738. doi: 10.3390/biom13050738 (PMC10216364; doi:10.3390/biom13050738)
Supplement: Supplementary file 1 [file biomolecules-13-00738-s001.zip › supplementary materials.pdf]

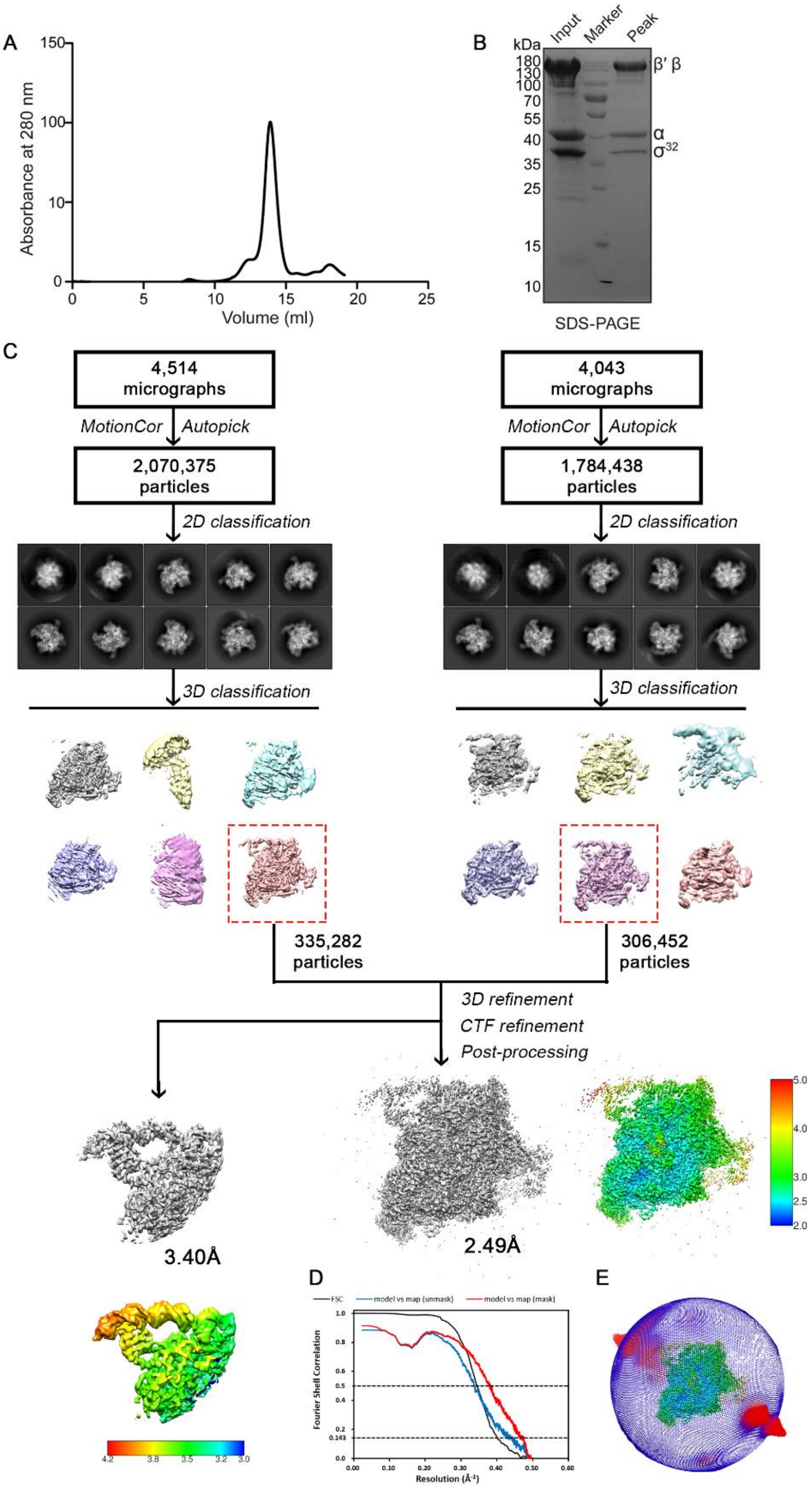

Figure S1. Purification of *E. coli* σ^{32}-RPo complex and Cryo-EM of the *E. coli* σ^{32}-RPo complex. (A) Chromatogram map of gel filtration. (B) SDS-PAGE of the purified complex. (C) Pipeline of the cryo-EM data processing. (D) The Fourier shell correlation (FSC) analysis. The dotted line represents the 0.143 FSC cut off. The final resolution is determined at 2.49 Å. (E) The angular distributions of *E. coli* σ^{32}-RPo particle and Density map for *E. coli* σ^{32}-RPo, colored by local resolution.

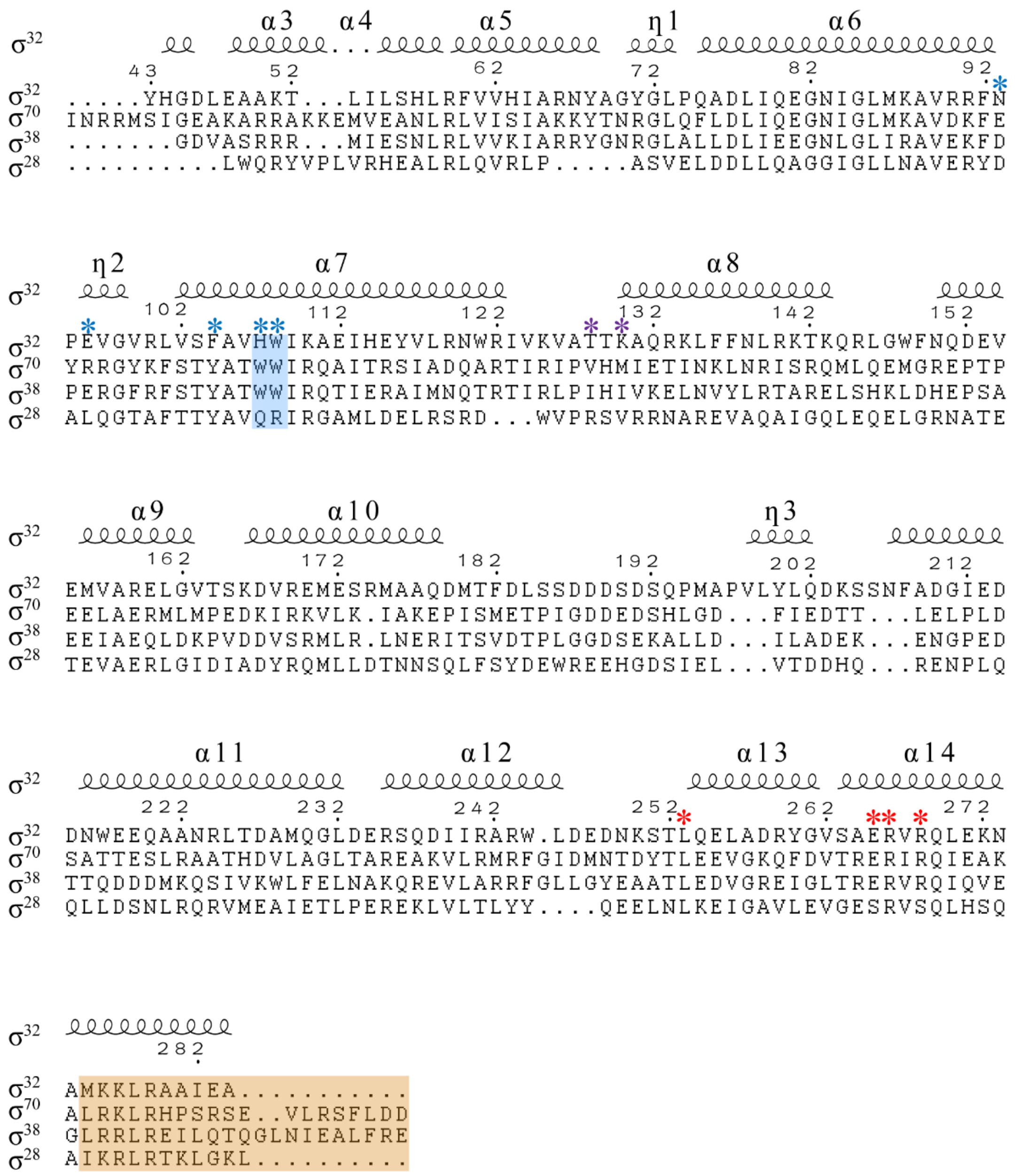

Figure S2. Sequence alignment of σ factors. Sequence alignment analysis between σ^{32}, σ^{70}, σ^{38}, σ^{28}. The experimentally validated residues in σ_4, σ_3, and σ_2 are indicated with red, purple and blue stars, respectively. The promoter unwinding residues in σ_2 are shown in light blue frame. The replaced region in the σ_4 tail of σ^{38}, σ^{28} is shown in light brown frame, consistent with diagrams in Figure 3E.

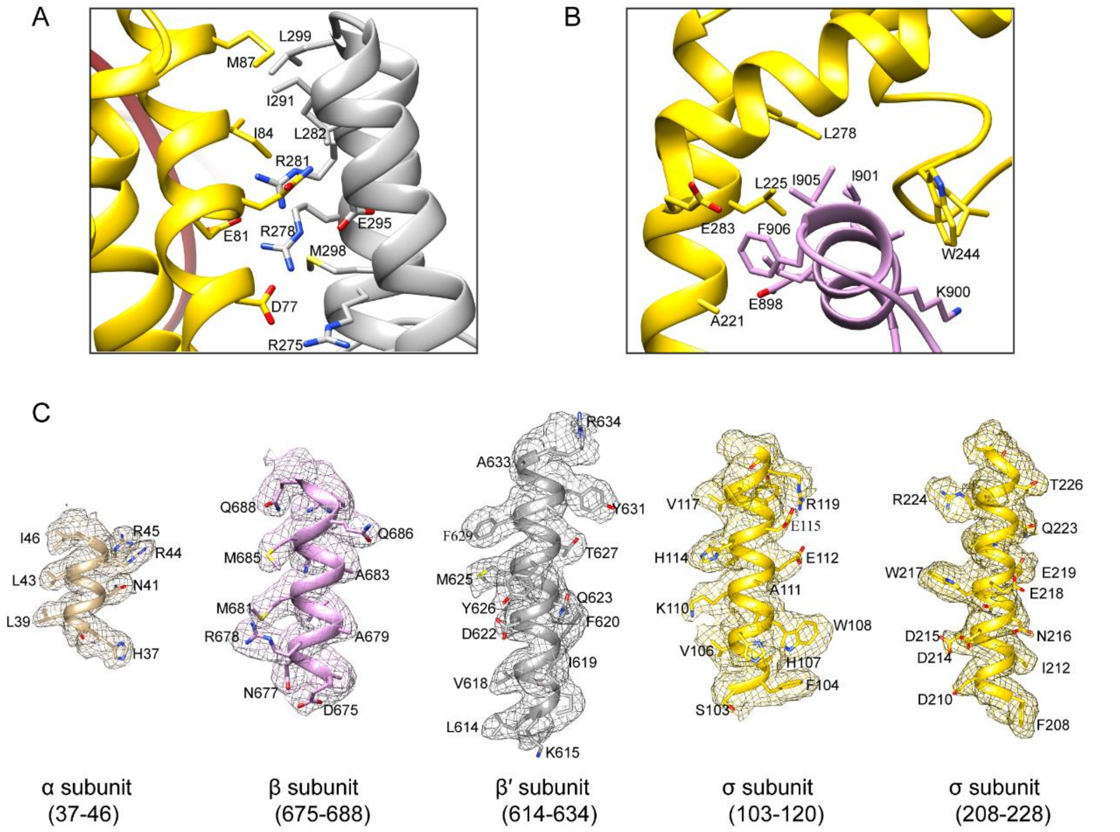

Figure S3. Structural and interacted details of pointed motifs. (A) The interaction details between σ_2 and β' clamp helix. (B) The interaction details between σ_4 and βFTH. (C) The structural details in α, β, β', and σ^{32} motifs.

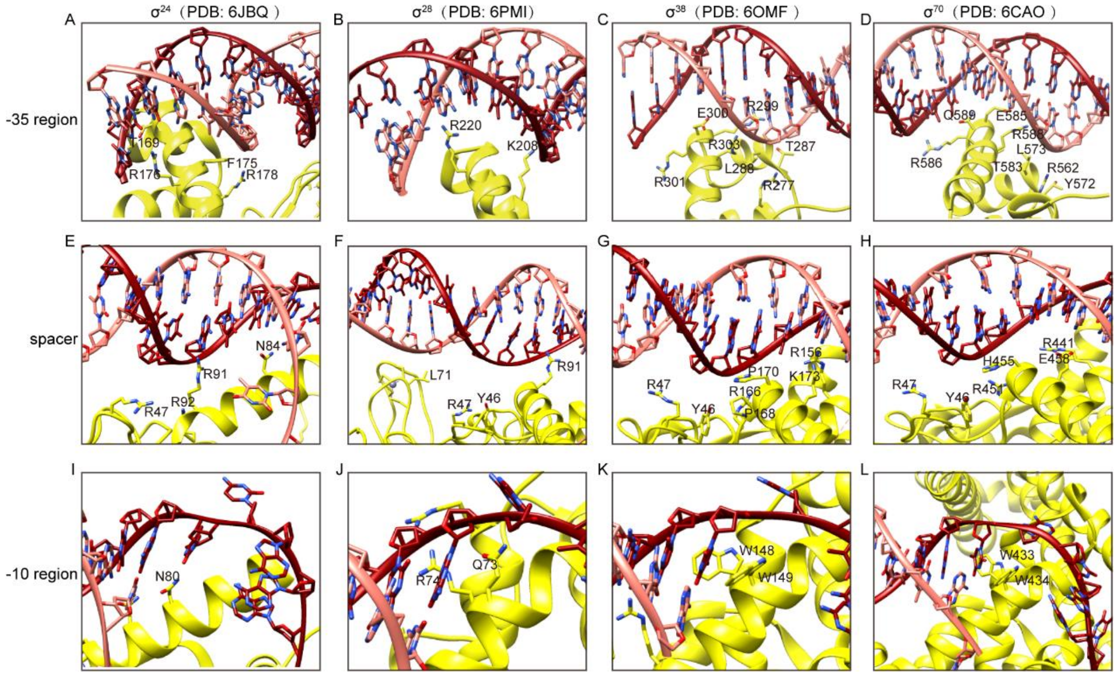

Figure S4. The detailed interactions between σs (σ^{24}, σ^{28}, σ^{38} and σ^{70}) and corresponding promoters. (A-D) The interaction details between σ_4 (σ^{24}, σ^{28}, σ^{38} and σ^{70}) and corresponding -35 elements. (E-H) The interaction details between σ_3 (σ^{24}, σ^{28}, σ^{38} and σ^{70}) and corresponding spacer regions. (I-L) The wedge residues in different σs (σ^{24}, σ^{28}, σ^{38} and σ^{70}).

Table S1. Kinetic parameters from Figure. 2C of RPo formation by *E. coli* RNAP holoenzyme comprising wild-type or derivatives of *E. coli* σ^{32}.

**Samples**	a_1	$k_{obs,1}$ (s^{-1})	a_2	$k_{obs,2}$ (s^{-1})
Ec holo σ^{32} (WT)	1.719±0.008	0.1102±0.0007	2.819±0.003	0.0107±0.0004
Ec holo σ^{32} (V97A)	1.532±0.006	0.0362±0.0009	2.596±0.003	0.0069±0.0003
Ec holo σ^{32} (N94A)	1.446±0.007	0.0911±0.0005	2.361±0.002	0.0104±0.0006
Ec holo σ^{32} (H107A)	1.179±0.006	0.0242±0.0015	2.426±0.002	0.0063±0.0005
Ec holo σ^{32} (F104A)	0.969±0.005	0.0244±0.0018	2.077±0.002	0.0061±0.0002
Ec holo σ^{32} (W108A)	0.895±0.004	0.0599±0.0011	1.556±0.001	0.0079±0.0002
Ec core	0.155±0.058	0.0136±0.0038	0.289±0.001	0.0047±0.0003

Table S2. Kinetic parameters from Figure. 2D of RPo formation by *E. coli*

**Samples**	a_1	$k_{obs,1}$ (s^{-1})	a_2	$k_{obs,2}$ (s^{-1})
Ec holo σ^{32} (WT)	1.770±0.010	0.1199±0.0033	2.773±0.003	0.0144±0.0007
Ec holo σ^{32} (WW)	1.819±0.009	0.1424±0.0022	3.019±0.003	0.0122±0.0006
Ec holo σ^{32} (QR)	1.122±0.006	0.0899±0.0006	1.675±0.002	0.0079±0.0005
Ec core	0.156±0.002	0.0148±0.0002	0.289±0.001	0.0015±0.0003

RNAP holoenzyme comprising wild-type or derivatives of *E. coli* σ^{32}.
