# Supplementary figures and images for: Structural Insight into the Mechanism of σ32-Mediated Transcription Initiation of Bacterial RNA Polymerase
Source: Biomolecules. 2023 Apr 25;13(5):738. doi: 10.3390/biom13050738 (PMC10216364; doi:10.3390/biom13050738)

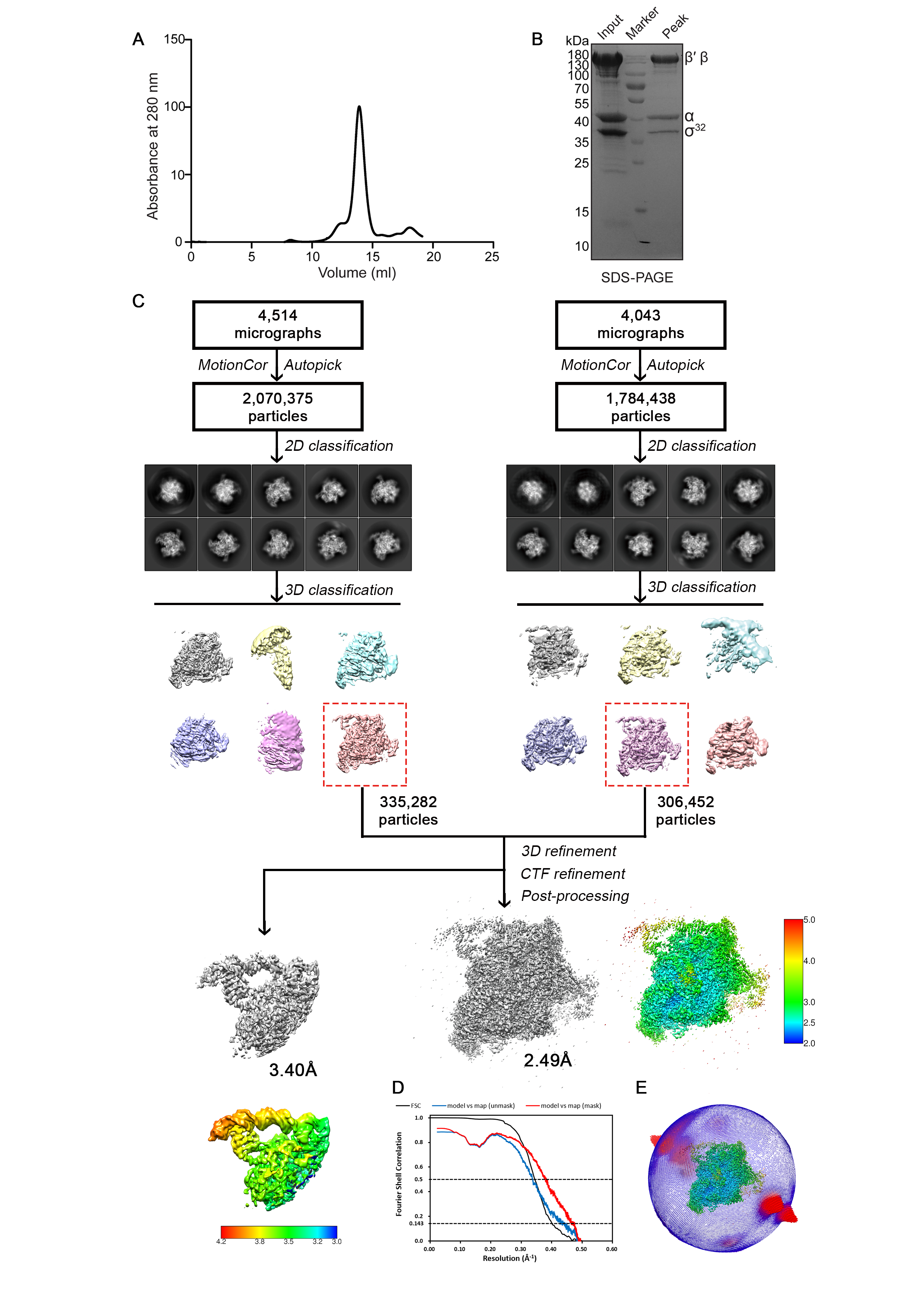

Supplement: Supplementary file 1 [file biomolecules-13-00738-s001.zip › F. S1.tif]

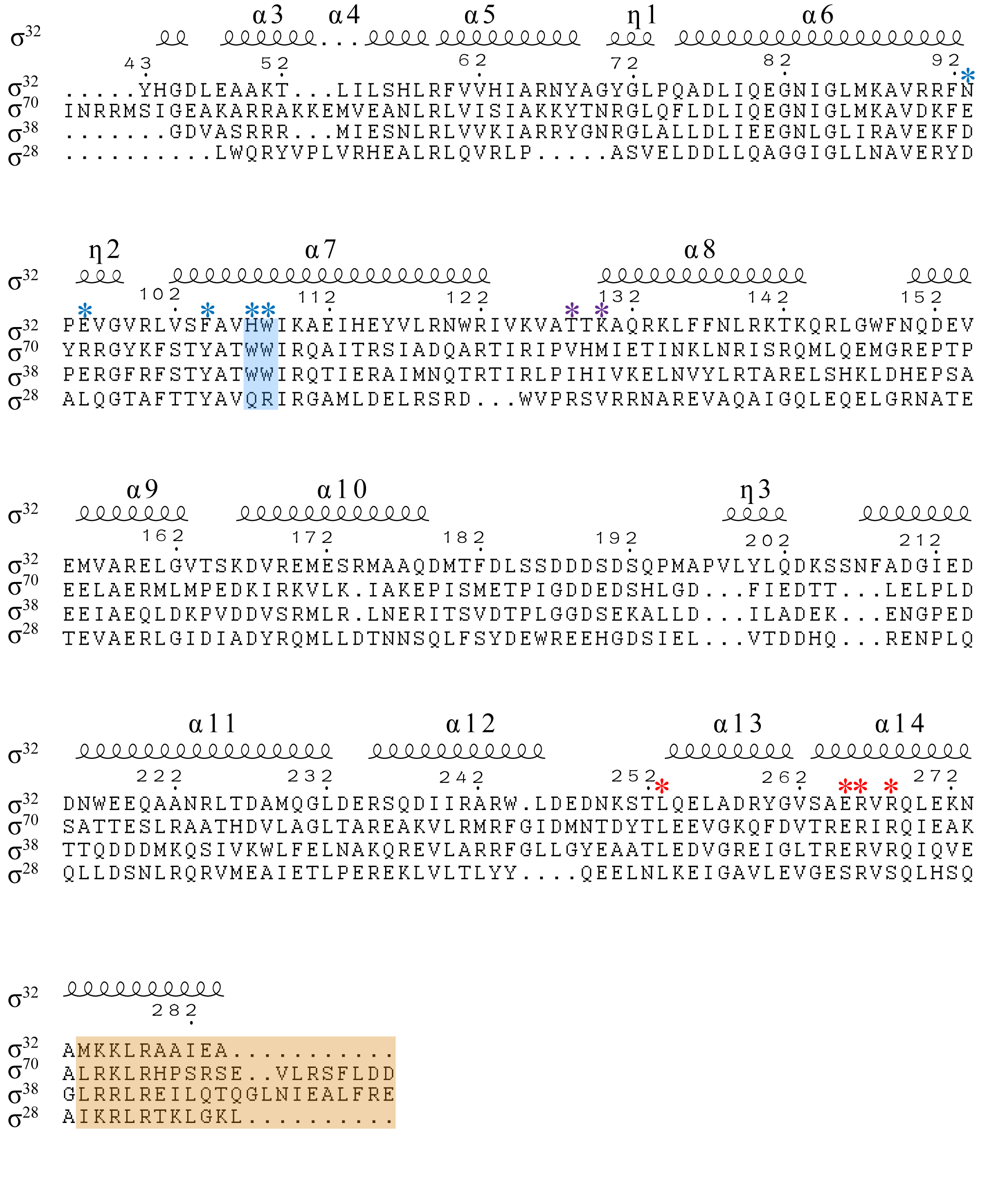

Supplement: Supplementary file 1 [file biomolecules-13-00738-s001.zip › F. S2.tif]

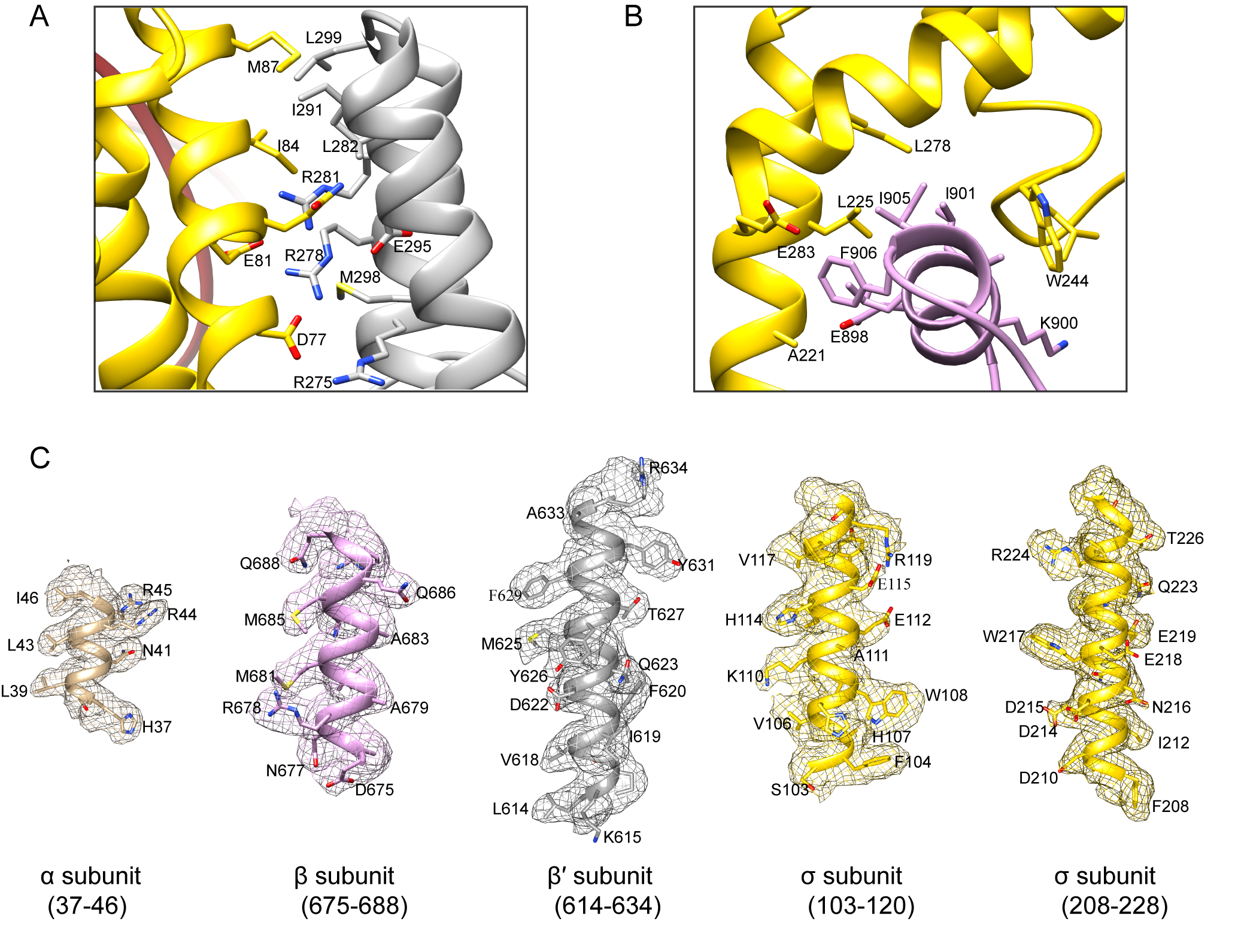

Supplement: Supplementary file 1 [file biomolecules-13-00738-s001.zip › F. S3.tif]

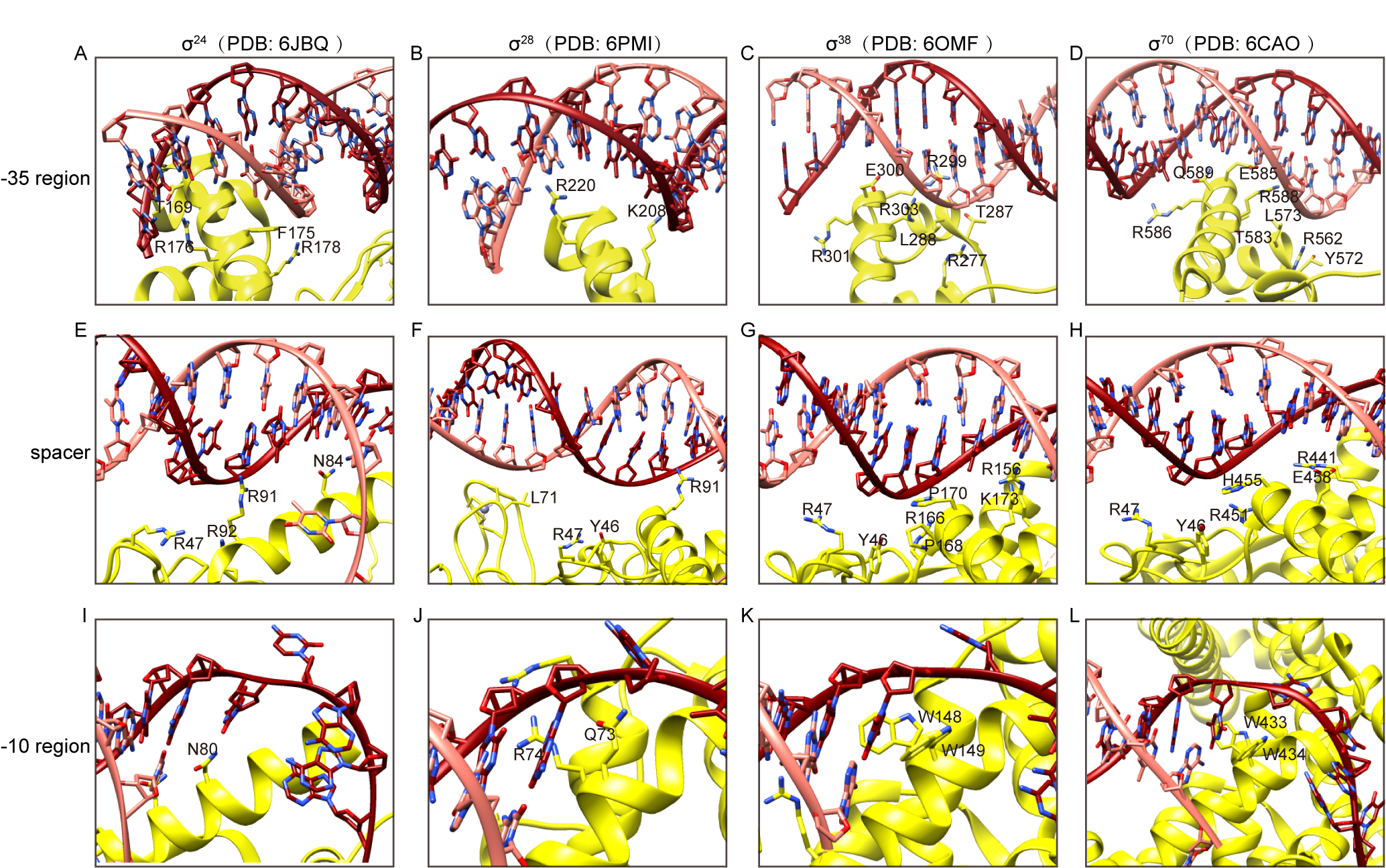

Supplement: Supplementary file 1 [file biomolecules-13-00738-s001.zip › F. S4.tif]
